# Spontaneous brain state oscillation is associated with self-reported anxiety in a non-clinical sample

**DOI:** 10.1038/s41598-020-76211-1

**Published:** 2020-11-12

**Authors:** Lei Qiao, Xi Luo, Lijie Zhang, Antao Chen, Hong Li, Jiang Qiu

**Affiliations:** 1 Shenzhen Key Laboratory of Affective and Social Cognitive Science, School of Psychology, Shenzhen University, Shenzhen, 518060 China; 2 Key Laboratory of Cognition and Personality, Ministry of Education, Department of Psychology, Southwest University, Chongqing, 400715 China

**Keywords:** Emotion, Human behaviour

## Abstract

The anti-correlation relationship between the default-mode network (DMN) and task-positive network (TPN) may provide valuable information on cognitive functions and mental disorders. Moreover, maintaining a specific brain state and efficaciously switching between different states are considered important for self-regulation and adaptation to changing environments. However, it is currently unclear whether competitions between the DMN and TPN are associated with negative affect (here, anxiety and depression) in non-clinical samples. We measured the average dwell time of DMN dominance over the TPN (i.e., the average state duration before transition to another state, indicating persistent DMN dominance) with a sample of 302 non-clinical young adults. Subsequently, we explored individual differences in this persistent DMN dominance by examining its correlations with subjective depression and anxiety feelings. Moreover, we linked state transition between DMN/TPN dominance with right fronto-insular cortex (RFIC) blood oxygen-level dependent signal variability. We found that the average dwell time of DMN dominance was positively associated with self-reported anxiety. Furthermore, state transition between DMN or TPN dominance was positively linked to RFIC activity. These findings highlight the importance of investigating the complex and dynamic reciprocal inhibition patterns of the DMN and TPN and the important role of the RFIC in the association between these networks.

## Introduction

There has been increasing interest in the macro-scale functional organization of the brain, particularly in two spatially distinct and temporally anti-correlated networks: the “default-mode” and “task-positive” networks (DMN and TPN, respectively)^1–4^. Coherent fluctuations can be found in the DMN regions during the resting state, while these regions show a deactivated response (mostly exhibit sub-baseline signal deflections) to a series of cognitive and attention-demanding tasks^5–7^. DMN activity has been considered to represent internally directed, self-reflective processes, and the brain activates the DMN when it is not occupied by a specific stimulus-dependent or resource-demanding task^8^. While the DMN (also called the task-negative network) is commonly anti-correlated with regions belonging to the TPN, which includes a set of regions generally activated during the performance of external attention-demanding tasks. The TPN appears to be associated with task-related patterns of elevated alertness and has also been correlated with response preparation and selection^9–11^. Several studies have found that abnormal activation or deactivation of the DMN and TPN was associated with neuropsychiatric and physiological disorders^12–15^.

Crucially, the anti-correlation between the DMN and TPN (reciprocal inhibition between the DMN and TPN) can be viewed as switching balance between attention to internal self-related thoughts and feelings and external stimuli and tasks^16^. Internally directed self-focused thoughts are characterized by increased activation in the DMN and decreased activation in the TPN, whereas externally oriented attention is characterized by decreased activation in the DMN and increased activation in the TPN^8^. In addition, the correlated and anti-correlated resting-state functional brain networks have been shown to be robust and reliable^17–20^. Recently, it was shown that the anti-correlation strength of the DMN and TPN can provide valuable information on cognitive functions as well as mental disorders^8,21^. For instance, intra-individual variation of the correlation between the DMN and TPN regions was associated with cognitive performance in a flanker task^22^ and a working memory task^23^. Intriguingly, the anti-correlation relationship of the DMN and TPN regions has also been related to mental disorders such as schizophrenia^24^, attention deficit hyperactivity disorder^25^, and autism^26^.

Importantly, abnormal DMN or TPN activity has been reported in individuals with anxiety and depression. For instance, Sheline et al.^27^ revealed that in patients with depression, DMN activity during active reappraisal and passive looking at negative stimuli was not reduced. Moreover, Cooney et al.^28^ examined the neural correlation of rumination in healthy individuals and those with depression using a rumination induction task; they found that DMN regions such as the medial prefrontal cortex (mPFC) and the posterior cingulate cortex (PCC) showed enhanced activation in participants with depression compared with healthy controls. Resting-state functional magnetic resonance imaging (fMRI) studies have found similar results in participants with depression, showing that DMN dominance over the TPN was linked to individual differences in rumination symptoms^29,30^. Abnormal DMN activity, including less pronounced mPFC deactivation and greater PCC deactivation, has been found in patients with anxiety^31^. Furthermore, Forster et al.^32^ revealed that trait anxiety was related to decreased recruitment of TPN regions during a sustained attention task.

Recently, increasing attention has been paid to the dimensional aspect of psychopathology, which suggests that some psychiatric disorders are characterized by a clinical continuum spanning from minor symptoms in healthy subjects, to those with subthreshold clinical symptoms, to people with manifest disorders. Affective disorders are especially likely to show continuity of severity from mild and/or transient worry or sadness to more severe symptoms^33,34^. Among affective disorders, depressive and/or anxious symptoms are prevalent in non-clinical populations and thus might exhibit a form of continuity from non-clinical to clinical samples^35,36^. This spectrum of mental disorders suggests the importance of studying non-clinical populations for prevention, early detection, and intervention of psychological disorders; yet, few biological studies have examined non-clinical subjects^34,37^. Moreover, exploring minor or subthreshold depressive and/or anxious symptoms in non-clinical subjects could reduce potential medication and treatment confounding, which is frequently observed in clinical populations, and provide clues about factors associated with resilience or compensatory changes^36^.

Previous studies have suggested that the antagonistic relationship between the DMN and TPN might generate two distinct modes: one characterized by DMN activity, which is a self-referential and introspective state, and the other involving TPN activation, which is an extrospective state^16,38,39^. State oscillation between these two modes may reflect the underlying dynamics and organization of the brain^40^, while altered balance of these two states may contribute to unfavorable outcomes. Moreover, it has been suggested that the anti-correlation relationship of these two networks may be functionally more important than DMN or TPN activity per se^9,12,41^. Significantly, the ability to maintain a specific brain state and to switch between different states, which refers to “reliable patterns of brain activity that involve the co-activation and/or connectivity of multiple large-scale brain networks,” is important for self-regulation and adaptation to changing environments^42–44^. However, very few studies have investigated the correlation between brain state oscillation and non-clinical symptoms, and it is currently unclear whether competitions between brain networks are associated with negative affect (here, anxiety and depression) in non-clinical samples.

To examine whether resting-state oscillation between DMN and TPN activity may be associated with negative emotions such as depression and anxiety, we calculated the mean dwell time of the DMN and/or TPN dominance state (i.e., the average time a state lasts before transition to another state, indicating the persistence of DMN or TPN dominance) and the switching between the two with a large sample of non-clinical young adults^3,45^. We assumed that anxiety and depression might bias brain states toward a more persistent DMN-dominant state. Therefore, we examined the correlation of the mean DMN dwell time with the anxiety and depression scores. Furthermore, we calculated the association between DMN dwell time and blood oxygen-level dependent (BOLD) signal variability of the right fronto-insular cortex (RFIC), a region that is considered to be critically involved in switching between the DMN and TPN^46,47^. We hypothesized that more frequent transitions between the DMN and TPN would be associated with greater RFIC BOLD signal variability.

## Methods and materials

### Participants

In total, 302 right-handed, non-clinical young adults participated in the study as part of our ongoing project exploring the relationships between brain imaging and mental health^48^. The sample consisted of 157 (52%) women with a mean age of 19.73 years (SD = 1.22) and 145 (48%) men with a mean age of 20.25 years (SD = 1.31). Seven participants were excluded because of poor image data quality. In addition, participants were excluded if overall head motion was above 2 mm in translation and 2° in rotation, finally leaving 287 participants for subsequent analysis. All participants were recruited from the local community. All participants completed the Self-rating Depression Scale (SDS)^49^ and Self-rating Anxiety Scale (SAS)^50,51^; none had history of neurological or psychiatric illness. The study was approved by the Southwest University Brain Imaging Center Institutional Review Board. We obtained written informed consent from all participants. All methods were carried out in accordance with relevant guidelines and regulations.

### Measurement of anxiety and depression

The SDS^49^ and SAS^50^ were used to measure depression and anxiety levels, respectively^51^. The SDS contains 20 items measuring depressive symptoms and has shown satisfactory reliability and validity^51,52^. Each item is rated on a 4-point Likert scale with a response pattern ranging from 1 “a little of the time” to 4 “most of the time”^51^. Of the 20 items, 10 are worded positively and 10 negatively. A higher SDS score is indicative of a relatively greater level of depressive symptoms. In our study, the Cronbach’s alpha coefficient for internal consistency was 0.73.

The 20-item SAS is used to measure the frequency of anxiety symptoms in the latest week^50^, with each response rated on a 4-point scale from “none or a little of the time” to “most or all of the time.” This scale consists of 15 somatic (physiological) and five affective (psychological) symptoms that are commonly related to anxiety and have shown adequate internal consistency and test–retest reliability^51,53^. The SAS is considered a reliable and ecologically valid measure of subjective anxiety levels in patients as well as in non-clinical participants^53,54^. In our study, the Cronbach’s alpha coefficient for internal consistency was 0.73^54^.

### Data acquisition

All functional MRI images were obtained using a Siemens 3T Trio scanner (Siemens Medical Systems, Erlangen, Germany). A foam pad was used to minimize subject head motion. Resting-state fMRI images were acquired using a gradient echo type echo planar imaging sequence: repetition time/echo time = 2000 ms/30 ms, flip angle = 90 degrees, resolution matrix = 64 × 64, field of view = 220 × 220 mm, thickness = 3 mm, and acquisition voxel size = 3.4 × 3.4 × 4 mm. A total of 32 slices were used to cover the whole brain. Each session contained 242 volumes. Before the resting-state scanning, all subjects were instructed to relax and keep their eyes closed but not sleep^55^.

### Data preprocessing

The resting-state fMRI data were preprocessed using Data Processing Assistant for Resting-State fMRI^56,57^ implemented in the MATLAB 2009b (MathWorks, Natick, MA, USA) platform. The first 10 scans of each participant were discarded because the magnetization is in equilibrium and owing to subject adaptation to the scanning noise. The remaining 232 scans were slice-time corrected and subsequently realigned to the middle image to correct for head movement^55^. Next, all realigned images were spatially normalized to the Montreal Neurological Institute (MNI) template and resampled into a resolution of 3 × 3 × 3 mm; then, the images were smoothed with a 6-mm full-width at half-maximum Gaussian kernel to increase the signal-to-noise ratio^55^. The DMN and TPN were identified using a procedure according to Hamilton et al.^29^. The effect of low and high frequency physiological noise was reduced through bandpass filtering (0.01–0.08 Hz) and linear detrending^55^. The global mean, white matter, and cerebrospinal fluid signals were regressed to cancel the effects of non-neuronal BOLD fluctuations^9,58^. Global signal regression has been found to induce artificial anti-correlations and to distort the correlation patterns^59,60^. However, Fox et al.^61^ found that global signal regression increases anatomical specificity, thus supporting its usage (see also Chai et al.^62^). Given the studies indicating that resting-state networks are particularly susceptible to head motion^63,64^, we adopted the Friston 24-parameter model^65^ to regress out head motion effects from the realigned data based on the evidence that higher-order models show benefits in cancelling out head motion effects^66–68^. Functional connectivity was examined using the Resting-State fMRI Data Analysis Toolkit (REST) software package^69^.

### Identifying the DMN and TPN

**Figure 1:**
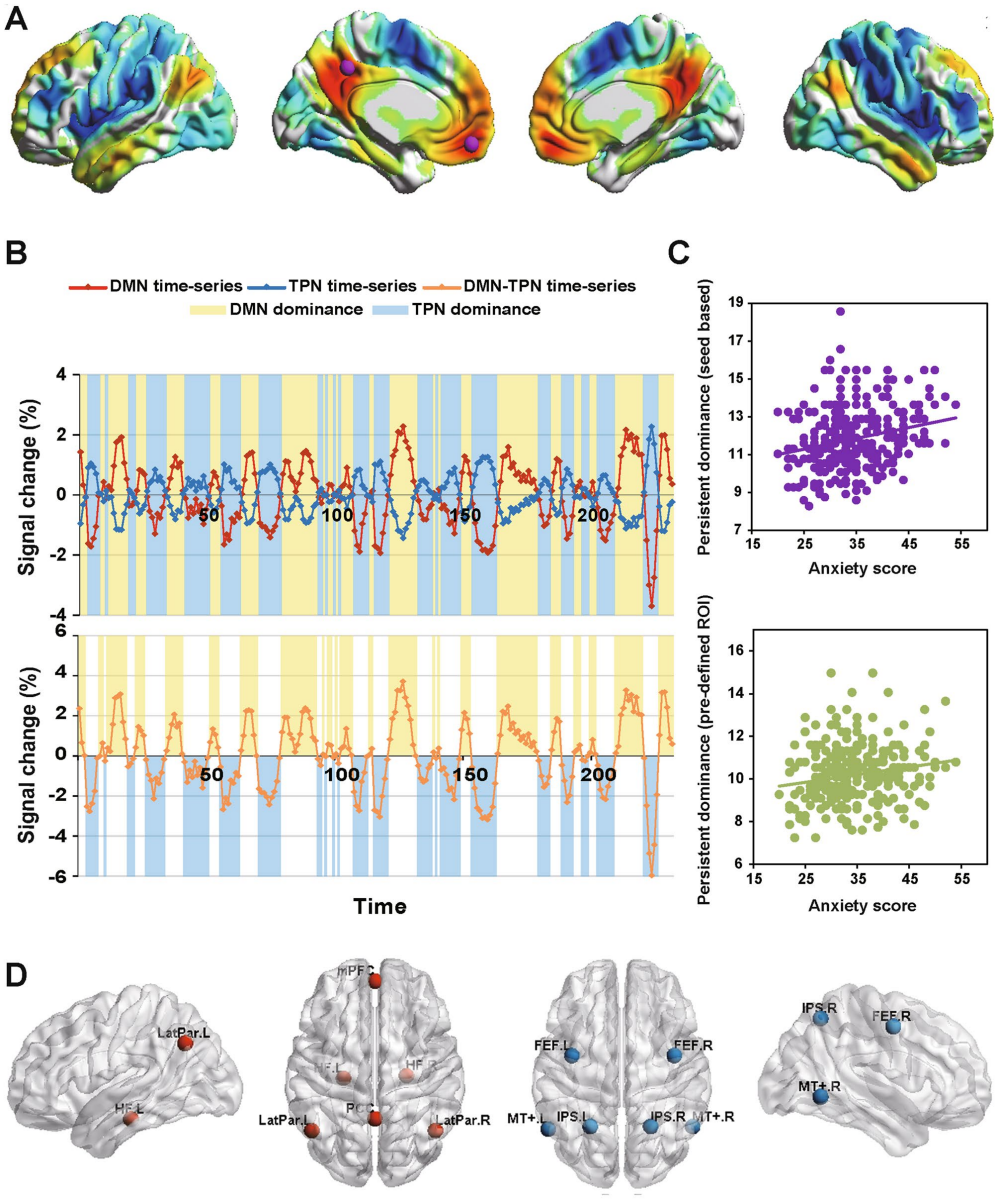
(**A**) The mPFC and PCC were selected to map the DMN (red) and TPN (blue) using a seed-based method. The brain networks were visualized using the BrainNet Viewer (https://www.nitrc.org/projects/bnv/). (**B**) The top figure presents the timeseries of DMN and TPN as well as their dominance; the bottom figure describes the timeseries of the DMN-TPN and the transition between DMN and TPN dominance. (**C**) Persistent DMN dominance was positively related to the anxiety score for both the seed-based network with global signal regression (top) and pre-defined network without global signal regression (bottom). (**D**) To extract the timeseries of DMN (red) and TPN (blue) without global signal regression, previously determined seed coordinates were used to define ROIs within these networks^71^. *DMN* default-mode network, *TPN* task-positive network, *ROI* region of interest, *PCC* posterior cingulate cortex, *mPFC* medial prefrontal cortex, *LatPar* lateral parietal cortex, *HF* hippocampal formation, *FEF* frontal eye field, *IPS* intraparietal cortex, *MT*+ middle temporal area, *L* left, *R* right.

As first described by Fox et al.^2^, we used a seed-region timeseries correlation analysis to derive the DMN and TPN. For identifying the DMN and TPN, (i) the seed region (Talairach coordinates) was placed at the mPFC and PCC (6-mm radius, centered at* x* = − 1,* y* = 47,* z* = − 4 and* x* = − 5,* y* = − 49,* z* = 40, respectively, Fig. 1A). The Talairach coordinates were first converted to the MNI space (mPFC,* x* = − 2,* y* = 51,* z* = − 14; PCC,* x* = − 7,* y* = − 46,* z* = 46) using the algorithm developed by Lancaster et al.^70^. The WFU Pickatlas toolbox (Wake Forest School of Medicine, Winston Salem) was used to create a binary mask image containing the two regions of interest (ROIs). The image was then resliced into 3 × 3 × 3 mm. Subsequently, the timeseries of the ROIs were extracted by averaging the timeseries of all voxels within the mask. Note that the timeseries of the two ROIs were extracted from the noise-covariate corrected dataset and averaged into a single timeseries^29^. (ii) We used the REST software to calculate the correlation between the seed-region timeseries data and whole-brain, preprocessed voxel timeseries data. (iii) The correlation coefficients were converted to the *z*-score using Fisher’s *r*-to-*z* transformation to improve the normality of the partial correlation coefficients. (iv) One-sample *t*-test was performed to identify the DMN and TPN. The DMN was defined as the set of regions whose timeseries data were positively correlated with the averaged timeseries of the mPFC–PCC seed (Fig. 1A). Similarly, the TPN was defined as the set of regions whose timeseries data were negatively correlated with the averaged timeseries of the mPFC–PCC seed regions (voxel level *p* < 0.000001, cluster threshold = 20 voxels). (v) Binary masks of the identified DMN and TPN were prepared using the xjview toolbox (https://www.alivelearn.net/xjview/)^51^. (vi) The masks of the DMN and TPN were applied to each participant’s data to extract the BOLD signal from the noise-covariate corrected data.

Given the controversy of removing the global signal in the preprocessing step of R-fMRI data^59,61,72^, we performed an additional analysis that did not regress out the global signal^71,73^. All other preprocessing steps were the same as the data preprocessing steps mentioned above. To extract the timeseries of the DMN and TPN, previously determined seed coordinates were used to define the ROIs within these networks^71^. The coordinates of the DMN and TPN were obtained from previous studies^19,71^. The centers of each ROI of the DMN and TPN regions are listed in Table 1. Several 6-mm radius sphere ROIs centering on the seed coordinates were created for the DMN and TPN, respectively. (A) Two binary masks of the DMN and TPN were created using the WFU Pickatlas toolbox. (B) The images were then resliced into 3 × 3 × 3 mm. (C) The masks of the DMN and TPN were applied to each participant’s data to extract the BOLD signal from the noise-covariate corrected data; note that the BOLD time courses within each ROI were averaged for the DMN and TPN^74^. We found similar results as those of the global signal regressed data (Fig. 1C).

**Table 1:** Anatomical regions used to define the default-mode and task-positive networks of the brain.

Region	Abbreviation	L/R	Network	MNI Coordinates		
				*x*	*y*	*z*
Medial prefrontal cortex	mPFC	med	D	0	52	− 6
Posterior cingulate cortex	PCC	med	D	0	− 53	26
Lateral parietal cortex	LatPar	L	D	− 48	− 62	36
		R	D	46	− 62	32
Hippocampal formation	HF	L	D	− 24	− 22	− 20
		R	D	24	− 20	− 22
Frontal eye field	FEF	L	T	− 38	− 4	48
		R	T	40	− 4	48
Intraparietal cortex	IPS	L	T	− 24	− 58	52
		R	T	22	− 58	54
Middle temporal area	MT+	L	T	− 56	− 60	− 2
		R	T	54	− 58	− 4

*L* left hemisphere, *R* right hemisphere, *D* default-mode network, *T* task-positive network, *MNI* Montreal Neurological Institute.

### DMN and/or TPN dominance

The operational definition of DMN dominance or TPN dominance used here was similar to that used by Hamilton et al.^29^. Specifically, the timeseries data of the DMN and TPN were first extracted from the noise-covariate corrected dataset, resulting in two N-FRAME-long vectors for each subject. DMN dominance over the TPN was then defined by assigning a value of 1 for temporal frames when the DMN BOLD signal was greater than the TPN BOLD signal, and a value of 0 was assigned for temporal frames when the TPN BOLD signal was greater than the DMN BOLD signal^29^. Finally, the new vector was summed to produce an index of DMN dominance over the TPN for each participant. For more details regarding the validation of the metrics of DMN or TPN dominance, see Hamilton et al.^29^.

### Persistence and transition of the DMN/TPN dominance state

To characterize the temporal features of brain state oscillation, we calculated two metrics for each subject; the first metric comprised the average dwell time of DMN/TPN dominance, measured by the mean lifetime of DMN/TPN dominance (i.e., the mean time a state lasts before transition to another state), indicating the persistence of DMN or TPN dominance^3,45^; the other metric comprised the number of dominance state transitions^75,76^. Specifically, a 232-frame-long vector was derived by subtracting the TPN timeseries from the DMN timeseries for each subject. Subsequently, we counted the times where the new vector crossed over zero, representing transitions between DMN dominance and TPN dominance (Fig. 1B). Finally, the mean dwell time and state transitions of DMN/TPN dominance were calculated for each participant.

### State transitions of DMN/TPN dominance and right fronto-insular BOLD

We explored whether state transitions between DMN and TPN dominance were associated with RFIC BOLD signal variability, as this region is a key area responsible for switching between the DMN and TPN^46,47^. Specifically, we calculated BOLD signal variance (standard deviation, SD) of the whole brain voxel by voxel with the noise-covariate corrected data for each subject. Subsequently, Statistical Parametric Mapping-8 (SPM8, Wellcome Department of Cognitive Neurology, London, UK; https://www.fil.ion.ucl.ac.uk/spm) was used to calculate the correlation between the number of brain state transitions and the BOLD variance of brain regions. A box-shaped image mask corresponding to the RFIC region was constructed using MarsBar SPM Toolbox (https://www.sourceforge.net/projects/marsbar) and was applied to the brain maps (27 ≤ *x* ≤ 48, 0 ≤ *y* ≤ 28, and − 19 ≤ *z* ≤ 15)^29^.

### Brain–behavior correlation analysis

We examined the association between the average dwell time of DMN dominance and anxiety as well as depression. This correlation analysis was performed using SPSS 16.0 (SPSS Inc., Chicago, IL) by adding age and gender as nuisance covariates. In addition, to maintain the family-wise type-I error at *P* < 0.05, Bonferroni correction was used to adjust the significance threshold for the two calculated correlations (i.e., between the mean DMN dwell time and the SDS and SAS scores).

For further data analysis, we divided the participants into four different symptom groups (i.e., high depressed and high anxious, high depressed and low anxious, low depressed and high anxious, low depressed and low anxious) according to the mean SDS and SAS scores. Since there were significant differences in the number of subjects assigned to each group: the low depressed and high anxious group included the fewest subjects (33 subjects), we included 33 subjects for each group according to the ranking of SDS and SAS scores. Particularly, 33 subjects with the highest SDS and SAS rank were assigned to the high depressed and high anxious group; 33 subjects with the lowest SDS and SAS rank were assigned to the low depressed and low anxious group; 33 subjects in the low depressed and high anxious group were included who showed the maximum difference of SDS and SAS rank. Next, the persistent DMN/TPN dominance was submitted to 2 (high vs. low anxiety) × 2 (high vs. low depression) analysis of variance (ANOVA) with age and gender added as confounders.

To confirm that the association with the behavioral scores was indeed with the average DMN dwell time dominance and not with less pronounced deactivation of the DMN or greater activation of the TPN, the average deviation (AD) from zero of the DMN and TPN timeseries was added as a nuisance covariate. As an additional precaution, we calculated the AD from zero of the 232-frame-long difference vector (DMN time series − TPN time series) for each participant, representing the detachment magnitude of the DMN and TPN time series. The AD from zero of the difference vector and the anti-correlation coefficient between the DMN and TPN timeseries were also added as nuisance covariates.

Given the extensive comorbidity of anxiety and depression^77^, we systematically disentangled the association between persistent DMN dominance and anxious and depressive symptoms. Bivariate Pearson r (two-tailed) correlations also showed significant comorbidity of anxiety and depression (*r* = 0.60,* p* < 10^–27^). To ensure the specificity of anxiety and depression symptoms, we used partial correlations to control for depression symptoms when the association between DMN dwell time and anxiety scores was examined, and we partialled out anxiety symptoms when the association between DMN dwell time and depression scores was examined.

## Results

Table 2 shows the demographic and behavioral data of the participants.

**Table 2:** Demographic and behavioral data of the participants.

	Males (n = 138)	Females (n = 149)	*df*	*t*	*p*
Age (years)	20.30 (1.31)	19.74 (1.24)	285	3.67	0.00*
SAS score	34.49 (7.11)	34.16 (6.33)	285	0.51	0.61
SDS score	31.75 (6.00)	30.79 (6.13)	285	1.49	0.14

*SAS* Self-rating Anxiety Scale, *SDS* Self-rating Depression Scale.

*p < 0.01.

### The mean dwell time of DMN dominance was correlated with self-reported anxiety

**Figure 2:**
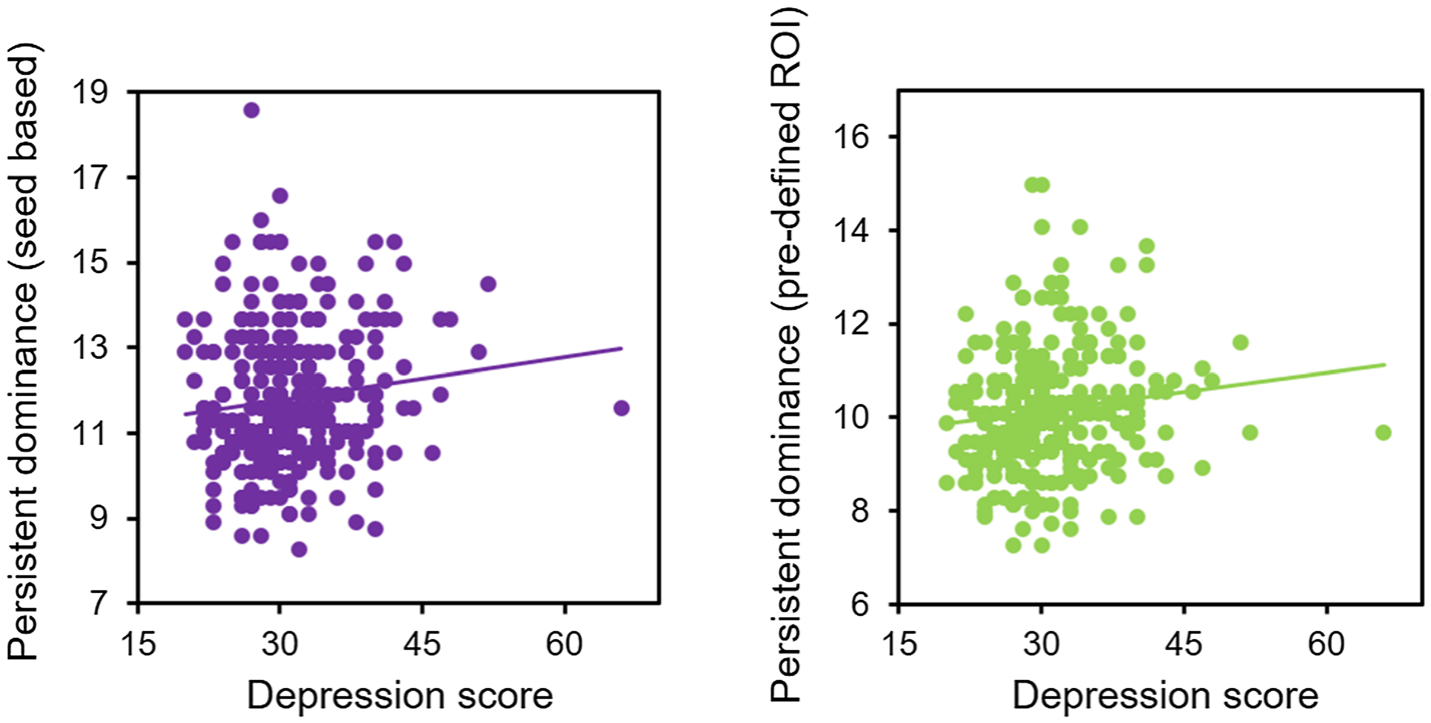
The relationship between persistent DMN dominance and depression score for both the seed-based network with global signal regression (left) and pre-defined network without global signal regression (right).

We found a significant positive association between the average dwell time of DMN dominance and the anxiety score; longer persistent DMN dominance was associated with higher anxiety scores,* r* (283) = 0.24,* p* < 10^–4^ (Bonferroni corrected) (Fig. 1C). The depression scores were also positively correlated with persistent DMN dominance,* r* (283) = 0.14,* p* < 0.05 (Bonferroni corrected). For the purpose of visual comparison, the relationship between persistent DMN dominance and depression scores is presented in Fig. 2. The results of the ANOVA were consistent with the correlation results: the main effect of anxiety on the persistent DMN/TPN dominance was significant, *F* (1,128) = 11.43, *p* < 0.005; the main effect of depression was also significant, *F* (1,128) = 4.04, *p* < 0.05; the interaction effect between anxiety and depression was not significant, *F* (1,128) = 0.28, *p* = 0.6 (n.s.).

When we treated the AD from zero of the DMN, TPN, and DMN-TPN timeseries, as well as the anti-correlation DMN and TPN coefficients as confounding covariates to partial out the effects of over/less activity of the DMN and TPN, the association between persistent DMN dominance and anxiety was also significant,* r* (279) = 0.25,* p* < 10^–4^ (Bonferroni corrected), as was also the association between persistent DMN dominance and depression,* r* (279) = 0.13,* p* = 0.052 (Bonferroni corrected). With this step, we confirmed that it was the persistent DMN dominance, rather than less pronounced deactivation of the DMN or greater activation of the TPN, that was associated with anxiety and depression scores. Furthermore, we calculated the partial correlations to control for depression symptoms when examining the association between persistent DMN dominance and anxiety,* r* (278) = 0.22,* p* < 0.001 (Bonferroni corrected). In contrast, the association between persistent dominance length and depression became nonsignificant when we calculated the partial correlations to control for anxiety symptoms,* r* (278) = 0.03,* p* = 0.68 (n.s.).

**Figure 3:**
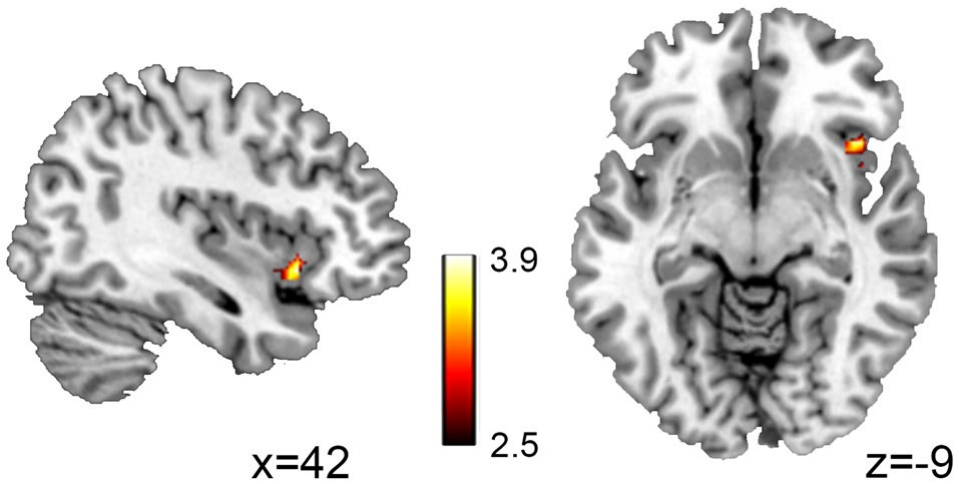
The sagittal and axial views of the significantly correlated RFIC are shown. The persistent DMN dominance was positively correlated with the BOLD signal variability of the RFIC,* p* < 0.05, corrected (peak MNI coordinate:* x* = 42,* y* = 18, z = − 9), the color bar represents the *t*-value. *RFIC* right fronto-insular cortex, *SD* standard deviation, *DMN* default mode network, *MNI* Montreal Neurological Institute.

In addition, we found that persistent DMN dominance was positively associated with RFIC BOLD signal variability (Fig. 3,* p* < 0.05, corrected). Finally, we explored the timeseries data without global signal regression using previously determined seed coordinates within the DMN and TPN and our result was replicated,* r* (283) = 0.20,* p* < 10^–3^ (see Fig. 1D for the seed ROIs).

## Discussion

In this study, we employed a novel method to measure persistent DMN dominance over the TPN (average dwell time of DMN dominance) and examined the relationship between persistent DMN dominance and anxiety and depression scores in non-clinical college students. Our data revealed that persistent DMN dominance was positively related to anxiety, that is, based on the results, individuals with more persistent DMN dominance appeared to have greater levels of self-reported anxiety. Moreover, we calculated the association between the state transition times and the variance of the RFIC BOLD signal. The results showed that persistent DMN dominance was positively correlated with RFIC BOLD signal variability (Fig. 3).

With respect to the anxiety domain, our result was consistent with those of previous studies. For example, Simpson et al.^78^ examined regional cerebral blood flow (BF) with positron emission tomography (PET). The authors found that BF decreases in DMN regions (especially the ventromedial PFC) were inversely correlated with anxiety self-rating even when no cognitive task was required. That is, the least anxious participants showed the largest BF reductions, while the most anxious participants exhibited no significant BF reduction. This finding provided strong evidence that BF reductions in the mPFC represent a dynamic balance of the brain between focused attention and subjective anxiety^79^. This dynamic balance may occur at a functionally active baseline or the default state of the brain^78^. Similarly, Zald et al.^80^ investigated the correlation between individual differences in negative affect (NA) and brain activity using PET. The results showed that resting regional cerebral BF within the DMN regions (especially the ventromedial PFC) was correlated with ratings of NA. Recently, it was revealed that healthy participants who exhibited greater DMN region activity during mindfulness meditation reported greater state anxiety^81^. In individuals with anxiety, abnormal hyperactivation of the DMN may result from hypersensitivity to salient self-relevant stimuli and higher post-event rumination^82^.

An increasing number of studies have found that anxiety can influence cognitive performance and/or cognitive control^83–87^. For instance, Ansari and Derakshan^83^ demonstrated that anxiety can impair inhibitory control even in the absence of emotional stimuli, further supporting the notion that anxiety could modulate top-down attentional control whether or not threatening stimuli are present^88,89^. In our study, we found that shorter persistent DMN dominance was associated with less anxiety, possibly reflecting better regulation of self-referential thought processes and more flexible shift of attention from an introspective state to the external environmental. It is possible that participants experiencing lower levels of anxiety can easily suppress or eliminate the interference induced by negative emotional information (which requires TPN involvement to take effect) or they can easily stop the ceaseless worrisome thoughts and ruminations related to DMN region activity^30,90^, whereas the opposite pattern emerged in participants experiencing greater levels of anxiety. Therefore, inability to suppress DMN activity might result in reduced deactivation as well as more sustained activity of this network, which may reflect that the brain is dominated by more interoceptive awareness or ruminative internal thought. Consistent with this idea, previous studies have suggested that incomplete suppression of DMN activity is perhaps related to aberrant automatic emotional processing^91,92^.

Competition between the DMN and TPN regions is indicative of the antagonism between exogenous and endogenous loci of information processing. Significantly, it is proposed that a critical function of cognitive control is to modulate this balance, by rapidly adjusting thoughts and behaviors according to changing internal states and varying external environments^93^. Furthermore, cognitive control can enhance mental flexibility by promoting goal-directed behaviors and suppressing irrelevant stimuli. Therefore, the present results may fit well with the attentional control theory (ACT)^89,94,95^. The ACT proposes that anxiety can influence top-down cognitive control. This theory also argues that anxiety-related worrisome thoughts would occupy and even use up cognitive resources, thus interfering with cognitive control. In addition, it is also assumed that this resource competition mechanism particularly impairs processing efficiency; compensatory mechanisms are needed to maintain performance effectiveness by increasing effort. Therefore, according to this theory, the ability to downregulate negative emotions might be impaired^91,96^.

However, anxiety has been linked to a neural correlate of increased reactive control, i.e., increased transient activity and reduced sustained activity of the BOLD signal, during the n-back working memory task in the cognitive control network^85^. At first glance, the present findings seem to be pointing to the opposite direction, since our result showed that increased sustained and reduced transient activity was associated with a higher level of self-reported anxiety. However, upon further examination, our results do not contradict those of the above-mentioned study because they were derived from resting-state data. Further, anxiety may be associated with impaired capacity to maintain cognitive task goals in working memory, as this capacity is occupied by a sustained internal attentional focus toward worry, rumination, and other forms of negative self-referential processing or by an external focus toward unforeseeable threats in the environment^97^, both of which may be related to more sustained activity during the resting state. In this case, if we regard the resting-state BOLD signal as the baseline, the subtraction of the task condition timeseries and resting-state baseline may produce the same results as those of the study mentioned above (i.e., increased transient activity and reduced sustained activity).

It has been proposed that the relative activity of the DMN and TPN may be controlled by specific brain regions, most likely the RFIC^47,98^. In addition, the fronto-insula is involved in task initiation, attention maintenance, and performance monitoring^43,99^. Recently, Anderson et al.^98^ explored the gradients of connectivity between the DMN and TPN. They found that the anterior insula was strongly anti-correlated with the core regions of the DMN. Importantly, the anti-correlation between this region and the DMN may be particularly important for cognitive control^100^. Notably, in our study, we found that persistent DMN dominance was positively related to RFIC BOLD signal variability, which was contrary to our assumption. This result possibly reflects that it is more difficult for “DMN-hijacked” individuals to flexibly shift their attention from interoceptive events to the external environment, and compensation by increased RFIC engagement is required.

In conclusion, we measured the antagonistic inhibition patterns between the DMN and TPN by the mean dwell time of DMN dominance over the TPN and explored the persistent and transition natures of the DMN/TPN dominance state. We found that persistent DMN dominance was positively correlated with individual anxiety scores. Moreover, state transition between DMN or TPN dominance was positively linked to RFIC activity. These results highlight the antagonistic feature between the DMN and TPN and provide new insight into elucidating the association between functional brain networks and anxiety.
